# In vitro mineral apposition analysis of two Colombian plant extracts on Amelogenesis imperfecta teeth

**DOI:** 10.1002/cre2.485

**Published:** 2021-09-09

**Authors:** Sandra J. Gutiérrez‐Prieto, Luis G. Sequeda‐Castañeda, Gabriela M. Penedo‐Jaramillo, Andrea V. Chacín‐Nieto, Daniel R. Contreras‐Cáceres, Gloria C. Moreno‐Abello, María P. Galvis‐Rincón, Fredy O. Gamboa‐Jaimes, Pilar E. Luengas‐Caicedo

**Affiliations:** ^1^ Department of Dental Systems, Dentistry Research Centre, School of Dentistry Pontificia Universidad Javeriana Bogotá Colombia; ^2^ Department of Chemistry, Faculty of Sciences Pontificia Universidad Javeriana Bogotá Colombia; ^3^ Department of Pharmacy, Faculty of Sciences Universidad Nacional de Colombia Bogotá Colombia; ^4^ Department of Periodontal Systems, Dentistry Research Centre, School of Dentistry Pontificia Universidad Javeriana Bogotá Colombia; ^5^ Department of Microbiology, Faculty of Sciences Pontificia Universidad Javeriana Bogotá Colombia

**Keywords:** Amelogenesis imperfecta, Colombia, medicinal plant, mineral apposition

## Abstract

**Objective:**

To determine if native Colombian *Piper marginatum* Jacq. and *Ilex guayusa* Loes plant extracts have a remineralizing effect on teeth with Amelogenesis imperfecta in comparison with the commercial products Clinpro‐3M and Recaldent™.

**Material and Methods:**

An in vitro study was carried out with 128 human teeth slices (64 healthy and 64 with Amelogenesis imperfecta) on which an initial Raman spectroscopy was performed followed by Raman spectroscopies at 0, 24, 48, and 72 h to determine possible remineralization by observing mineral increase or decrease as a result of *P*. *marginatum* Jacq. and *I*. *guayusa* Loes extract application in comparison to control substance (Clinpro and Recaldent™) application. Obtained data were analyzed using a bivariate method with a *t* unidirectional test. Significant differences among groups were determined by an ANOVA with Dunnett post hoc tests.

**Results:**

Native *I*. *guayusa* Loes and *P*. *marginatum* Jacq. Colombian plants extracts exhibited phosphate and orthophosphate mineral apposition, where *P*. *marginatum* Jacq. presented better results.

**Conclusions:**

Native Colombian *I*. *guayusa* Loes and *P*. *marginatum* Jacq plant extract might in the future be useful for dental tissue remineralization, as they induced phosphate and orthophosphate mineral apposition, main components of tooth enamel. These types of natural compounds can become an alternative to fluorine, whose ingestion is harmful to the human body.

## 1 INTRODUCTION

Amelogenesis imperfecta (AI) are hereditary disorders that affect tooth enamel quality and quantity, causing dental sensibility, susceptibility to cavities, fracture, and even tooth loss. Four phenotypes are known: Hypoplastic type, whose main distinction is a thin enamel with grooves. Hypomaturation or type II, presenting enamel protein accumulation, with translucent appearance and occasional spots. Hypocalcified, characterized by enamel wear, resulting from decreased mineral content, up to 30% less enamel in comparison with non‐affected enamel; and hypomaturation/hypoplasia/taurodontism or Type IV (Hu et al., 2007; Hurtado et al., 2015; Witkop Jr., 1988; Wright et al., 1993). At present, treatment for these defects are limited to restoration, which demands further elimination of dental tissue dental and use of fluoride and substances (America, 2020; ClinPro, 2015; Fan et al., 2011; Li et al., 2014; Petzold, 2001; Shen et al., 2020) to remineralize these tissues (Oliveira et al., 2011).

Enamel remineralization includes mineral incorporation, such as carbonate, magnesium, sodium, fluoride, calcium and phosphate, among others. Generally, a direct association is observed between these ions in saliva and reduced tooth enamel demineralization (dental caries). These minerals actively intervene in remineralization, where calcium and phosphate are the most important in demineralization prevention. In tooth enamel, phosphate is found in greater quantities in comparison with calcium. In patients with poor dental hygiene and erosive agent consumption, a high cavity prevalence has been observed. Calcium does not seem to meet its remineralizing effect, thus in tooth enamel demineralization treatment fluorinated products have been a constant; even though their excessive use can be toxic, producing dental, and skeletal fluorosis (Li et al., 2014; Shen et al., 2020).

Fluoride varnishes are among the fluorinated products in the market, which are applied to the tooth's surface. Presently Clinpro (3M) is one of the most used. This product contains tricalcium phosphate in a patented formula based on fluoride, calcium, and phosphate, which is released in a continuous manner when in contact with saliva (ClinPro, 2015). Recaldent™ MI paste (GC) is another remineralization product with a patented formula. It is made of casein phosphopeptide (CPP) and amorphous calcium phosphate (ACP), proteins derived from milk that release calcium and phosphate in a bioavailable manner for its surface capture. CPP's main effect is early cavity remineralization, since this peptide contains elements that bind to calcium and stabilize calcium phosphate in an ACP solution. Among its limitations are lactose intolerance or allergies to milk products, thus this product cannot be administered to these patients. Furthermore, the time required to pursue a remineralizing effect is yet to be determined (America, 2020).

Another option to remineralize tooth enamel is by means of plant extracts, which are a natural alternative. Different studies have demonstrated *Galla chinensis* (a plant of Chinese origin), contains metabolites, such as tannins and flavonoids that remineralize tooth matrix. Its mechanism of action is not well known, but it has been determined that it promotes mineral apposition in cavity lesions in a safe manner. Rat in vivo studies have demonstrated this plant does not present toxic effects. Additionally, tooth enamel mineral penetration is deeper compared with fluoride which limits to the most superficial surface (Cheng et al., 2010; Chu et al., 2007; Zhang et al., 2016).

Colombia presents a wide variety of plant species, due to its geographical location. Thus, allowing for ethnobotany and medicinal plant studies in search of similar properties in native and endemic plants (Bussmann et al., 2018). Our previous studies describe several plant species in Colombia, including *Ilex guayusa* and *Piper marginatum* used by indigenous and rural communities for the treatment of oral cavity diseases (Bernal et al., 2011; García‐Barriga, 1992; Rojas et al., 2006; Sequeda‐Castañeda, 2021; Sequeda‐Castañeda et al., 2015; Sequeda‐Castañeda et al., 2016). *P*. *marginatum* Jacq. is present in the neotropic from Guatemala to Brazil and the Caribbean. Commonly known as “tooth healer,” its leaves are used in folk medicine to heal tooth aches, rheumatoid tumors, digestive problems, and skin wounds. Additionally, its stem is macerated and topically applied against tooth cavities (García‐Barriga, 1992; Mutis, 1997; Sequeda‐Castañeda et al., 2015). Phytochemical studies demonstrate the presence of steroids, alkaloids, flavonoids, phenolic compounds, phenylpropanoids and terpenoids, among others (Brú & Guzman, 2016). In rats, its oral administration (2 g/kg) did not induce a cytotoxic effect. On the contrary, parenteral route of administration (1 g/kg), did evidence piloerection, drooling, lacrimation, muscle relaxation, and dyspnea (Brú & Guzman, 2016; D'Angelo et al., 1997; García‐Barriga, 1992; Gonçalves et al., 2019; Gupta et al., 2008; Mutis, 1997; Reigada et al., 2007; Sequeda‐Castañeda et al., 2015).

Moreover, *I*. *guayusa* is present in the Western Amazon (Peru, Colombia, and Ecuador). It has a long ethnobotanical data, where it has been used as a mouthwash, herbal tea and energizing drink, among others (Lema‐Paguay et al., 2017; Sequeda‐Castañeda et al., 2016). Phytochemically it contains caffeine, triterpenes, chlorogenic acids, tannins, and flavonoids (Arteaga‐Crespo et al., 2020; Chianese et al., 2019; Dueñas et al., 2016; García‐Barriga, 1992; Gupta et al., 2008; Lema‐Paguay et al., 2017; Radice & Vidari, 2007; Sequeda‐Castañeda, 2021; Sequeda‐Castañeda et al., 2016;Wise & Negrin, 2020 ; Wise & Santander, 2018). Moreover, Racidi and Vidari (2007) reported presence of alkaloids, steroids, terpenes, and lactonic compounds (Radice & Vidari, 2007). Its toxicity in brine shrimp assays presented a LD_50_ greater than 10,000 μg/mL four aqueous and ethanol extracts. Guayusa consumption dates back to hundreds of years to the present and is registered as safe with health benefits (Wise & Negrin, 2020; Wise & Santander, 2018). Other reports have demonstrated the leaves of both plants have an estrogenic (Contero et al., 2018) and antibacterial effect against microorganisms responsible for periodontal disease (Contero et al., 2018; Gamboa et al., 2018).

Thus far, in vitro observations of metabolite apposition, such as tannins and flavonoids, on tooth enamel for remineralization (Epasinghe et al., 2015; Kharouf et al., 2020; Petti & Scully, 2009), and evaluation by means of different techniques including scanning electron microscopy, fluorescence microscopy, and X‐ray diffraction have been carried out (de Aza et al., 1997; Eberhardt et al., 2015; Sa et al., 2017). At present, Raman spectroscopy is presented as a noninvasive alternative technique to observe phenomena occurring directly in the oral cavity, because the emitted light does not affect the organism's tissues (Ramakrishnaiah et al., 2015; Sa et al., 2017). This technique employs the reflection of light generating spectra in the visible light range in the near infrared visible light or near ultraviolet, indicating and increase or decrease in minerals in the tissue of interest, through the intensity of a band or peak. The greater the intensity, the greater the presence of an element (Miyamoto et al., 2020). In the present study, Raman technique was employed to determine if *P*. *marginatum* and *I*. *guayusa* Colombian plant extracts were capable of mineral apposition on enamel of teeth with AI.

## 2 MATERIALS AND METHODS

### 2.1 Tooth sampling

This research was approved by the Pontificia Universidad Javeriana Dental School ethics and research committee (CIEFUJ, Minute No. 010 de 2012). Sixty permanent teeth indicated for exodoncy were collected from 38 patients between the ages of 20 and 32 years old after signed informed consent for donation of healthy tissues (teeth) by patients attending the school's odontological clinics. All 30 healthy teeth met the criteria of root and crown integrity without any restorations or cavities. Moreover, 30 teeth were collected from patients with AI with root integrity and without restorations or cavities.

### 2.2 Sample preparation

#### 2.2.1 Collection of teeth slices

After tooth extraction, each piece was thoroughly cleaned with saline solution to wash teeth and remove tissue remains. Following each tooth was cut in a coronal section and then in a sagittal section with a NTI‐Kahla New Technology Instruments® diamond blade leaving intact the tooth's enamel. Sagittal slices in control teeth, as well as AI teeth were 4 mm thick for a total of 128 slices, which were stored in 1.5 mL (Eppendorf®) tubes containing deionized water (free of microorganisms and mineral salts) or artificial saliva (Salivar, Farpag® laboratories). Slice distribution is described in Table 1: The control group (C) was made up of 64 slices of healthy teeth, of which 32 were stored in distilled water (C1) and 32 stored in artificial saliva (C2). The experimental group (E) was made up of 64 slices of teeth with AI, of which 32 were stored in distilled water (E1) and 32 stored in artificial saliva (E2).

**Table 1:** Tooth slice distribution from healthy individuals of patients with enamel defects (AI)

Teeth slices	Group	Storage medium
128	Control (C) 64 slices of healthy teeth	Water. 32 slices (C1)^a^
		Salivar. 32 slices (C2)^b^
	Experimental (E) 64 slices of teeth with enamel defect	Water. 32 slices (E1)^a^
		Salivar. 32 slices (E2)^b^

^a^ Deionized and sterilized water.

^b^ Artificial saliva from Farpag® Laboratory.

#### 2.2.2 In vitro simulations of enamel defects in control group C

Healthy control slices (C1) and (C2) were demineralized to induce enamel mineral loss, similar to the condition found in AI, with a hypocalcified phenotype due to the pathology (Wright et al., 1993). To model enamel defects samples underwent an aggressive acid etching based on the Buonocore technique (Buonocore, 1955; Buonocore, 1975). This procedure was carried out applying 3M phosphoric acid at 37% for 60 s, followed by a distilled water wash for 40 s, last samples were air‐dried.

#### 2.2.3 Teeth with enamel defects experimental group (*E*)

Experimental groups (E1) and (E2) corresponded to teeth slices with AI of the hypomineralized phenotype (Sabandal & Schäfer, 2016; Smith et al., 2017; Wright et al., 1993).

### 2.3 Application of remineralizing substances—Control and experimental groups

#### 2.3.1 Control group

The commercial products Clinpro (3M) and MI Paste, Recaldent™ (GC) were used as positive controls. In Colombia, these products are utilized by oral professionals to produce remineralization of tooth enamel. Application of these products was carried out following manufacturer's instructions. Clinpro (3M): A fine layer was applied with a micro‐brush on the tooth surfaces without rinsing (ClinPro, 2015). MI Recaldent™ Paste (GC): was applied with a brush on the tooth's clean surface, and allowed to adhere for 3 min. No rinsing after application was performed (America, 2020; Farooq et al., 2013).

#### 2.3.2 Experimental group

##### Extract acquisition

Plant material was collected in Mogambo, environmental trail, in the municipality of Viotá (Cundinamarca, Colombia). Plant species taxonomic identification was performed at the Pontificia Universidad Javeriana and Universidad Nacional de Colombian herbariums with voucher numbers HPUJ 28878 (*I*. *guayusa*) and COL575454 (*P*. *marginatum*), respectively, under the Access to Genetic Resources and their Derivative Products Contract No. 255 of 2019 of the Ministry of Environment and Sustainable Development (MinAmbiente). Total extracts were obtained by solid–liquid Soxhlet extraction and a 7:3 ethanol–water solvent mix using the plant:solvent ratio of (1:30), during 48 h. following, the extracts were concentrated by rotary evaporation at 40°C, until a dried extract was obtained. Dry extracts for each plant were prepared at 1 g/L concentration using as a solvent water or artificial saliva at 1:1 ratio under constant agitation at 50 rpm for 1 h. Extracts were applied on each tooth slice with a brush. After commercial product and plant extract application, teeth slices were stored again in properly labeled Eppendorf tubes containing distilled water or artificial saliva (Table 1). All samples were then placed in an automatic shaker (MaxQ 4000) at 95 rpm and 37°C, simulating the oral cavity environment (Sequeda‐Castañeda, 2021; Sequeda‐Castañeda et al., 2021).

### 2.4 Raman spectroscopy mineral apposition analysis

Raman spectroscopy was performed using ID Micro Raman spectrometer (Ocean optics), which employs ToupView and OceanView software for biological spectra analysis. Spectra were acquired at 70.3 mW with scanning times of 1, 5, and 10 s. Results are presented as the mean of three measurements per assayed time. An initial Raman spectroscopy was performed to each group before the application of any substance to determine baseline values. Following, to obtain reference spectra, spectroscopy was recorded for each substance: fluoride (Clinpro de 3M), MI Recaldent™ paste (GC), 37% phosphoric acid (3M), artificial saliva (Salivar®, Farpag® Laboratories), deionized water and both plant extracts (*I*. *guayusa* and *P*. *marginatum*). Last, Raman spectroscopy was recorded for each tooth slice to which substances were applied. For all groups vibrational spectra were observed at 955 and 1037.5 cm^−1^ for phosphate and orthophosphate, respectively, the main components of apatite (Ko et al., 2006; Ramakrishnaiah et al., 2015). Raman spectroscopy procedure was as follows: each sample was placed on a slide and placed in the spectroscope. A bean of light was impinged on a previously established point on the tooth's enamel and the reading was performed through one of the software's as described. ToupView allowed for surface inspection and OceanView permitted to read the spectra. Methodology is summarized in Tables 2 and 3.

**Table 2:** Sequence of procedures performed and substance exposure time for healthy teeth slices (Groups C1 and C2) with commercial products Clinpro, Recaldent™, and *Ilex guayusa* and *Piper marginatum* total extracts

	Storage medium	Activity					
		1	2	3	4	5	6
Healthy teeth (64 slices) Group C	Water (32 slices) C1	Initial Raman	Raman 0 h^a^	Clinpro (8)	Raman 24 h	Raman 48 h	Raman 72 h
				Recaldent™ (8)			
				*I*. *guayusa* (8)			
				*P*. *marginatum* (8)			
	Salivar (32 slices) C2	Initial Raman	Raman 0 h^a^	Clinpro (8)	Raman 24 h	Raman 48 h	Raman 72 h
				Recaldent™ (8)			
				*I*. *guayusa* (8)			
				*P*. *marginatum* (8)			

a Demineralized tooth.

**Table 3:** Sequence of procedures performed and substance exposure time for teeth slices with AI (Groups E1 and E2) with commercial products Clinpro, Recaldent™, and *Ilex guayusa* and *Piper marginatum* total extracts

	Storage medium	Activity				
		1	2	3	4	5
Teeth with enamel defect^a^ (64 slices) Group E	Water (32 slices) E1	Initial Raman (0 h)	Clinpro (8)	Raman 24 h	Raman 48 h	Raman 72 h
			Recaldent™ (8)			
			*I*. *guayusa* (8)			
			*P*. *marginatum* (8)			
	Salivar (32 slices) E2	Initial Raman (0 h)	Clinpro (8)	Raman 24 h	Raman 48 h	Raman 72 h
			Recaldent™ (8)			
			*I*. *Guayusa* (8)			
			*P*. *marginatum* (8)			

a Amelogenesis imperfecta.

### 2.5 Statistical analysis

Statistical analysis was performed using a bivariate analysis with a *t* unidirectional test. To determine multivariate significant differences a one‐way analysis of variance (ANOVA) was used. Furthermore, to determine significant differences among groups a Dunnett post hoc test was performed. Analyses were performed using STATISTICS software (Lesaffre et al., 2009).

In Table 2, an initial Raman spectroscopy was captured. A recording was performed after the demineralization treatment with 37% phosphoric acid, which corresponded to time 0, the following measurements are detailed (24, 48, and 72 h).

In Table 3, an initial Raman spectroscopy was captured on teeth with AI before applying any substance and after substance application.

## 3 RESULTS

### 3.1 Raman spectroscopy mineral apposition analyses in teeth slices

**Figure 1:**
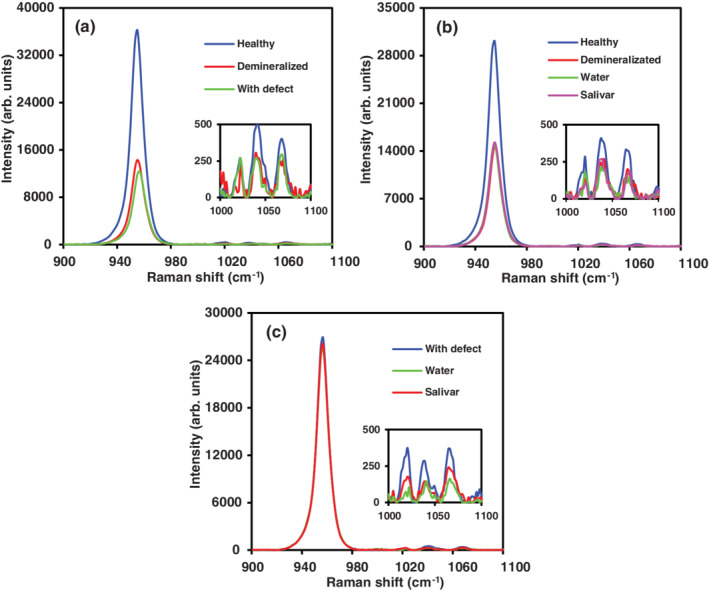
Raman spectra in healthy‐, demineralized‐, and teeth with enamel defects (a); healthy teeth and demineralized in contact with storage solution (b); teeth with enamel defect (AI) in contact with storage solution (c)

Raman spectra in teeth slices from healthy‐, demineralized‐, and teeth with enamel defects (AI) bands corresponding to phosphate and orthophosphate groups were observed at 955 and 1037.5 cm^−1^, respectively (Figure 1 **)**.

Raman spectra in healthy teeth presented a greater intensity for the phosphate (955 cm^−1^) and orthophosphate bands (1037.5 cm^−1^). However, when healthy teeth were demineralized Raman intensity decreased considerably. In teeth with AI, Raman intensity was lower in comparison with demineralized teeth, suggesting phosphate and orthophosphate mineral loss, due to the pathology responsible for tooth enamel defect (Figure 1a). No changes in intensity (arbitrary units) or in Raman shift (cm^−1^) were observed, when teeth were stored for 6 months at 4°C in water or artificial saliva (Figure 1b). In teeth with AI stored under the same conditions aforementioned, only a discrete change (reduction) was observed for Raman intensity corresponding to orthophosphates (1037.5 cm^−1^; Figure 1c), most likely due to hypomineralized tooth enamel, which is susceptible to mineral loss, due to its fragility by contact, wear, and decomposition (Buonocore, 1955; Buonocore, 1975; Miyamoto et al., 2020; Murrillo et al., 2015; Ramakrishnaiah et al., 2015; Sa et al., 2017).

**Figure 2:**
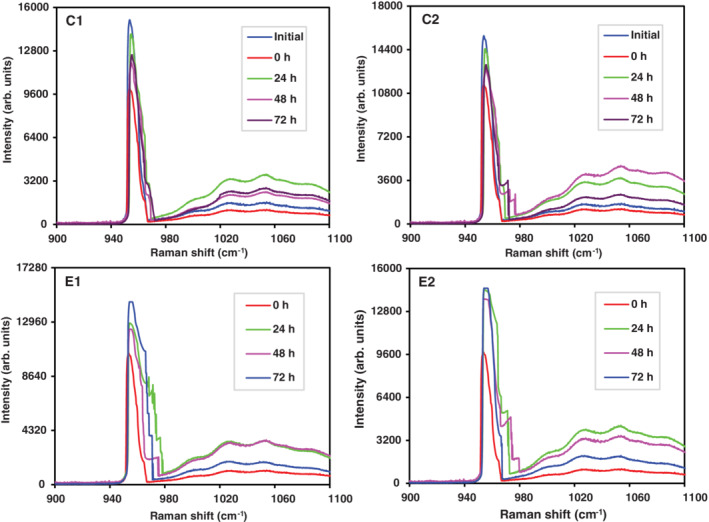
Raman spectra in monitoring phosphate (955 cm^−1^) and orthophosphate (1037.5 cm^−1^) mineral apposition using Clinpro on healthy demineralized teeth (C1: Stored in dH_2_O, C2; stored in salivar) and teeth with enamel defects (E1: Stored in dH_2_O, E2: Stored in salivar). In group C (C1 and C2), onset (initial) corresponds to teeth without demineralization and at time 0 h teeth were demineralized

Phosphate and orthophosphate mineral apposition was observed, when the commercial product ClinPro (3M) was used on demineralized teeth slices and those with AI (Figure 2). A greater Raman intensity was observed for phosphate apposition (955 cm^−1^) in demineralized teeth at 24 h, whereas in teeth with enamel defect Raman intensity increased with time of contact. Orthophosphate apposition at 1037.5 cm^−1^, demonstrated variability with times of exposure for demineralized teeth, as well as for teeth with AI. To determine significant differences, the corresponding statistical analysis was performed (Section 3.2). Qualitatively, the commercial product Clinpro presented a better mineral apposition in teeth with AI and healthy teeth undergoing a demineralization process (artificial cavities).

**Figure 3:**
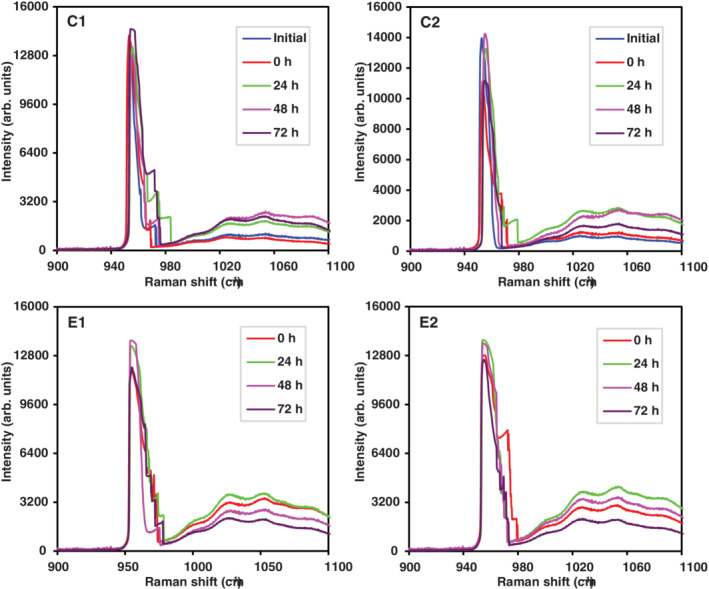
Raman spectra in monitoring phosphate (955 cm^−1^) and orthophosphate (1037.5 cm^−1^) mineral apposition using *Piper marginatum* total extract on healthy demineralized teeth (C1, C2) and teeth with tooth enamel defects (E1, E2). In group C, onset corresponds to teeth without demineralization and at time 0 h teeth were demineralized

Raman spectra for teeth slices treated with total *P*. *marginatum* are illustrated in Figure 3. In demineralized teeth, a mineral apposition effect was observed for phosphate and orthophosphate. In teeth with AI a greater phosphate mineral apposition was identified between 24 and 48 h, whereas for orthophosphate it was observed at 24 h. Variability was statistically evaluated (Section 3.2).

**Figure 4:**
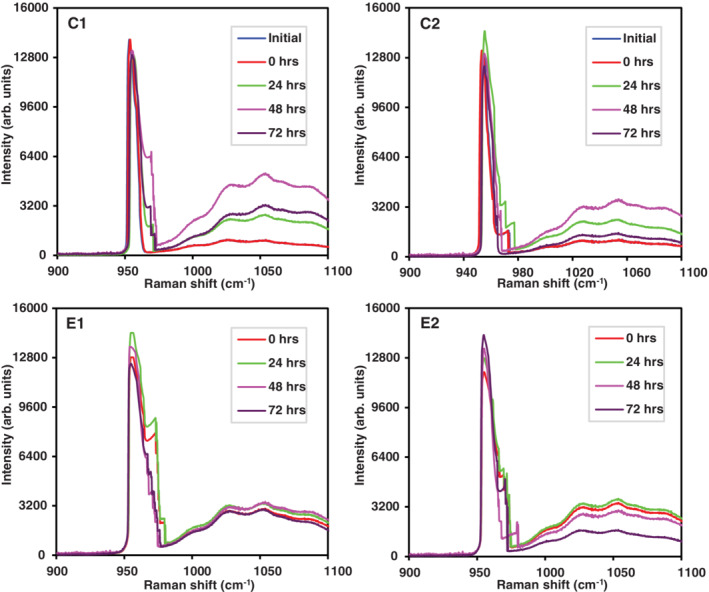
Raman spectra in monitoring phosphate (955 cm^−1^) and orthophosphate (1037.5 cm^−1^) mineral apposition using *Ilex guayusa* total extract on healthy demineralized teeth (C1, C2) and teeth with tooth enamel defects (E1, E2). In group C, onset corresponds to teeth without demineralization and at time 0 h teeth were demineralized

*Ilex guayusa*'s Raman spectra to visualize extract mineral apposition effect are depicted in Figure 4. Variability was observed in demineralized teeth, where at 24 h a greater phosphate apposition was observed and at 48 h this effect was evidenced for orthophosphate. In teeth with AI, phosphate and orthophosphate apposition was greater at 24 h. Likewise, variability was observed among samples, thus to determine significant difference a statistical analysis was performed (Section 3.2).

**Figure 5:**
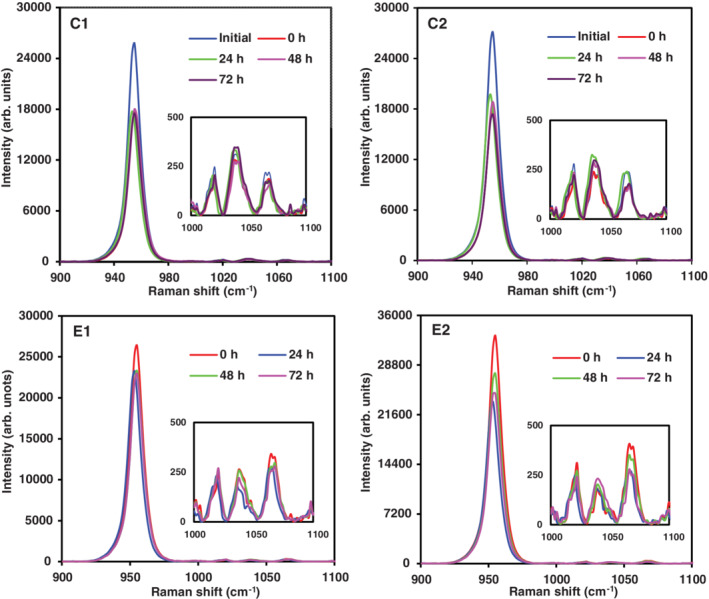
Raman spectra in monitoring phosphate (955 cm^−1^) and orthophosphate (1037.5 cm^−1^) mineral apposition using Recaldent™ on healthy demineralized teeth (C1, C2) and teeth with tooth enamel defects (E1, E2). In Group C, onset corresponds to teeth without demineralization and at time 0 h teeth were demineralized

Registered spectra for Recaldent's™ effect on phosphate and orthophosphate mineral apposition are shown in Figure 5. A decreased apposition was observed in comparison with Clinpro and total extract effect on phosphate and orthophosphate mineral apposition for demineralized teeth and teeth with AI. In the present work, qualitative data demonstrated Recaldent™ commercial product did not have the expected mineral apposition result, thus it was not utilized as a positive control. Hence, the commercial product Clinpro was selected as a remineralizing agent. Statistical analyses were performed among Clinpro and *I*. *guayusa* and *P*. *marginatum* total extracts.

### 3.2 Statistical analysis

Data obtained for phosphate and orthophosphate are illustrated in Tables 4 and 5, respectively.

**Table 4:** Raman intensity multivariate analysis of phosphate^a^ apposition in teeth with Clinpro, *Piper marginatum*, and *Ilex guayusa* total extract application

Extract or control	Tooth type	Storage medium	Raman intensity (arb. units)			
			Initial^b^	Apposition (h)		
				24	48	72
Clinpro	Healthy	Water	11321.6 ± 2003.1	13639.6 ± 2003.3	13125.3 ± 2076.2	12778.8 ± 2794.3
		Salivar	11214.0 ± 4510.8	14461.8 ± 2341.3	12787.1 ± 3695.5	13118.7 ± 5299.1
	With defect^c^	Water	NA	12873.4 ± 1416.4	12382.3 ± 1241.9	14559.7 ± 1491.3
		Salivar	NA	14401.0 ± 402.2	13723.0 ± 1523.9	14537.6 ± 9025.4
*Piper marginatum*	Healthy	Water	13712.6 ± 908.3	9216.5 ± 1140.6	10685.5 ± 1921.4	14318.7 ± 2313.2
		Salivar	10208.2 ± 3032.3	12522.6 ± 806.2	13689.8 ± 1672.4	10996.2 ± 2566.2
	With defect^c^	Water	NA	13283.2 ± 4738.2	13787.9 ± 2490.5	11518.5 ± 6932.9
		Salivar	NA	13813.5 ± 1259.6	13601.0 ± 3824.7	12382.0 ± 5107.2
*Ilex guayusa*	Healthy	Water	13408.9 ± 1869.9	6975.4 ± 519.6	9381.0 ± 1647.7	10107.6 ± 3309.0
		Salivar	12685.3 ± 2929.8	13837.8 ± 1221.4	12950.4 ± 2638.0	12096.4 ± 3879.0
	With defect^c^	Water	NA	13125.0 ± 3124.9	13343.1 ± 1143.3	12226.8 ± 4379.1
		Salivar	NA	11824.8 ± 4095.2	11934.1 ± 3057.6	13647.5 ± 2313.2

Abbreviation: NA, not applied.

^a^ (*n* = 8). 955 cm^−1^ Raman shift for phosphates.

^b^ Demineralized tooth.

^c^ Amelogenesis imperfecta.

**Table 5:** Raman intensity multivariate analysis of orthophosphate^a^ apposition in teeth with Clinpro, *Piper marginatum*, and *Ilex guayusa* total extract application

Extract or control	Tooth type	Storage medium	Raman intensity (arb. Units)			
			Initial^b^	Apposition (h)		
				24	48	72
Clinpro	Healthy	Water	1418.2 ± 927.7	3022.7 ± 1796.4	2964.3 ± 1568.1	2375.5 ± 971.2
		Salivar	1221.5 ± 497.5	3785.2 ± 1225.8	4756.7 ± 1616.9	2412.2 ± 1360.7
	With defect^**c**^	Water	NA	3522.1 ± 861.3	3520.5 ± 1390.1	1806.8 ± 427.0
		Salivar	NA	4222.8 ± 1432.5	3475.3 ± 1584.5	2004.3 ± 821.6
*Piper marginatum*	Healthy	Water	863.3 ± 391.8	1959.9 ± 1488.5	2569.4 ± 1367.4	2258.9 ± 1333.5
		Salivar	1216.1 ± 787.5	2814.1 ± 1427.7	2686.2 ± 772.8	1792.0 ± 1116.7
	With defect^c^	Water	NA	3763.9 ± 1124.3	2670.1 ± 1723.8	2052.0 ± 1298.3
		Salivar	NA	4197.6 ± 1207.2	3517.6 ± 1311.1	2038.2 ± 977.1
*Ilex guayusa*	Healthy	Water	926.5 ± 317.6	2539.0 ± 1264.6	5144.3 ± 2740.9	3136.4 ± 1532.0
		Salivar	1117.4 ± 396.6	2391.4 ± 838.1	3710.0 ± 1212.2	1536.6 ± 651.9
	With defect^c^	Water	NA	3332.6 ± 852.4	3425.9 ± 1366.1	2940.2 ± 1858.9
		Salivar	NA	3649.1 ± 1003.8	2912.6 ± 1193.0	1688.6 ± 808.9

Abbreviation: NA, not applied.

^a^ (*n* = 8). 1037.5 cm^−1^ Raman shift for orthophosphates.

^b^ Demineralized tooth.

^c^ Amelogenesis imperfecta.

#### 3.2.1 Phosphate

The control group (C1 and C2) made up of demineralized healthy teeth in 37% phosphoric acid did not present significant differences between teeth slices stored in distilled water and artificial saliva (*p* > 0.05). In regard to applied substances, ClinPro (3M), *I*. *guayusa*, and *P*. *marginatum* total extracts at 24, 48, and 72 h it was observed Clinpro demonstrated the greatest phosphate apposition in comparison with total extracts in a significant manner (*p* = 0.000). Moreover, no significant differences were observed between extracts (*p* = 0.509, *p* = 0.239, and *p* = 0.071 for each of the evaluated time points).

With reference to the experimental group (teeth with AI, E1, and E2) the only significant difference observed was for phosphate apposition in teeth slices stored in distilled water and artificial saliva (*p* = 0.047). Concerning phosphate apposition with applied substances at 24, 48, and 72 h, it was observed Clinpro presented significantly higher phosphate apposition in comparison with plant extracts (*p* = 0.000). In contrast, no significant differences were recognized between extracts with *p* values of *p* = 0.821 for 24 h, *p* = 0.087 for 48 h, and *p* = 0.369 for 72 h for each time point.

Multivariate analysis demonstrated *I*. *guayusa* and *P*. *marginatum* extract compared with Clinpro at 24, 48, and 72 h presented significant differences (*p* < 0.001), where the highest apposition was observed at 24 h and the lowest at 72 h. At 24 h, no significant differences were observed among substances, type of tooth slice or storage media. Clinpro presented higher apposition in comparison to *I*. *guayusa* and *P*. *marginatum* in demineralized teeth stored in distilled water. *I*. *guayusa* and *P*. *marginatum* presented significant differences at 48 h (*p* = 0.013). However, at 24 and 72 h no significant differences were observed (*p* = 0.621, *p* = 0.06, respectively).

#### 3.2.2 Orthophosphate

Bivariate analysis in control group with demineralized healthy teeth (C1 and C2) revealed no significant differences were present between teeth slices stored in distilled water and those in artificial saliva (*p* > 0.05). Relating to substances at 24, 48, and 72 h no significant differences were observed among Clinpro and the extracts, nor between plant extracts (*p* > 0.05).

Likewise, in the experimental group with AI teeth slices (E1 and E2) no significant differences were observed between slices stored in distilled water and artificial saliva (*p* > 0.05). No significant differences were identified among Clinpro and plant extracts for the evaluated time points (*p* > 0.05), except for Clinpro and *I*. *guayusa* at 24 h, where ANOVA analysis revealed significant differences (*p* < 0.05), with Clinpro presenting the highest mineral apposition. Furthermore, no significant differences were observed between extracts at 24, 48, and 72 h (*p* > 0.05).

#### 3.2.3 Recaldent™

This product was not used as a remineralization positive control, because in comparison with Clinpro and total plant extracts, mineral apposition effect was lower. Thus, a comparative study of this product with others was not carried out, and result analysis was only bivariate. Findings revealed phosphate at 0 and 48 h presented significant differences between teeth slices stored in distilled water and artificial saliva (*p* < 0.05). In addition, significant differences were identified between demineralized control teeth and AI at 24, 48, and 72 h, *p* = 0.02, *p* = 0.000, and *p* = 0.001, respectively.

Concerning orthophosphate, it was evidenced at 0, 48, and 72 h significant differences were observed between healthy demineralized teeth stored in distilled water and artificial saliva (*p* < 0.001, *p* < 0.05, *p* < 0.05, respectively). Moreover, significant differences were detected between healthy demineralized teeth and teeth with enamel defects at 24 h (*p* < 0.05) and 72 h (*p* < 0.001).

Figure 5 depicts Raman spectra for phosphate and orthophosphate obtained for Recaldent™ treatment in control and experimental groups.

Data demonstrated (Table 4) all substances contributed to phosphate apposition in healthy teeth demineralized with 37% phosphoric acid and in teeth with AI at 24, 48, and 72 h.

Results exhibited significant differences for orthophosphate apposition at 24 h between *I*. *guayusa* and Clinpro, the latter being the one with the highest apposition.

## 4 DISCUSSION

In the present in vitro study, slices from demineralized healthy teeth and teeth with a hypocalcified phenotype from AI patients were obtained (Gutierrez, 2013; Hurtado et al., 2015; Murrillo et al., 2015). The following commercial products ClinPro® (3M‐ESPE) and Recaldent™ were applied to teeth slices; these products are utilized by health professionals to remineralize tooth enamel (America, 2020; ClinPro, 2015). Furthermore, *I*. *guayusa* and *P*. *marginatum* plant extracts from native Colombian plants were also applied to enamel as a natural alternative. The objective of this study was to determine by means of Raman spectroscopy if the use of this natural alternative could apposition mineral on tooth enamel comparatively to fluoride (ClinPro).

ClinPro (3M) commercial product is categorized as a strong fluoride source, effective in mineralization processes (Lendenmann & Bolis, 2018). At present fluoride is still one of the most utilized products for dental caries treatment, and as a remineralizing agent (Clinpro, 2017; Mohd Said et al., 2017). However, its high concentration and long exposure during enamel formation, results in dental fluorosis manifested as a mottled patches and fragile enamel (González‐Cabezas & Fernández, 2018). At the cellular level, fluoride causes a rupture in the cell's membrane with organelle injury, such as the Golgi apparatus, endoplasmic reticulum, nucleolus, and mitochondria (DenBesten & Li, 2011; Gu et al., 2020; Zuo et al., 2018). Therefore, as a less toxic alternative various products based on calcium and phosphate have been used for mineral apposition on tooth enamel (America, 2020; Indrapriyadharshini et al., 2018; Zero, 2009), as well as plant species. To date, various reports point out to the enamel remineralization effect in lesions, such as caries by gallotannins in *Galla chinensis* (Chinese native plant) (Chu et al., 2007; Huang et al., 2017). The plant's gallotannin metabolite seems to act in the generation of a complex between the metabolite and the tooth's enamel matrix, forming a calcium transporter that facilitates calcium apposition on the tooth's enamel (Cheng et al., 2010; Farooq et al., 2013; Huang et al., 2010; Huang et al., 2017; Zhang et al., 2016).

In the present study, *I*. *Guayusa* Loes and *P*. *marginatum* Jacq. were selected among 200 Colombian native plant species (initial phase performed by our research group) because of their use in pathologies related to the oral cavity in folk medicine (indigenous and rural communities) (Bernal et al., 2011; Sequeda‐Castañeda, 2021). The aforementioned plants contain secondary metabolites associated with a possible remineralization effect in comparison to that reported for *Galla chinensis* (Zhang et al., 2015; Zhang et al., 2016). Therefore, the aim of this work focused on determining whether the plant extracts used were capable of mineral apposition on teeth with enamel defect, such as AI. In this hereditary pathology, different phenotypes are present, where tooth enamel quality and quantity are affected, in a severe or mild presentation, in deciduous or permanent teeth (Sabandal & Schäfer, 2016; Smith et al., 2017).

In this research, *I*. *guayusa* and *P*. *marginatum* total plant extracts were applied on tooth slices of teeth with AI (experimental group). Likewise, for the control group healthy teeth slices were collected, to which a demineralization process was carried out. Two commercial products were applied: 3M's Clinpro and Recaldent™ MI Paste (GC), which are frequently used by dentists to remineralize enamel (Tables 2 and 3). Raman spectroscopy was used to assess if plant extracts appositioned minerals on tooth slices with enamel defects. This technique is based on probing the symmetric valence vibration near 960 cm^−1^ as a marker for crystallinity for phosphate and 1037.5 cm^−1^ for orthophosphate. Therefore, elements, molecules, compounds, or material vibrational states can be evaluated (Jones et al., 2019; Tsuda & Arends, 1993). In 2019, Guentsch et al. used Raman spectroscopy to investigate the effect of a mineralization kit on tooth enamel (Guentsch et al., 2019). In the present study, the minerals used as the basis for apposition analysis on dental slices were phosphate and orthophosphate. These minerals make part of hydroxyapatite, the main compound of tooth enamel (He et al., 2020). Our group has previously used and implemented this technique using teeth slices with artificial caries (Sequeda‐Castañeda, 2021; Sequeda‐Castañeda et al., 2021).

In this study, results obtained from Raman spectroscopy demonstrated fluoride varnish (Clinpro 3M), used as a positive control (gold standard), allowed for greater phosphate release and mineral apposition in the control group (demineralized healthy teeth; Figure 2), as well as the experimental group (teeth with AI) compared with *I*. *guayusa* and *P*. *marginatum* plant extracts (*p* = 0.000), Figures 3 and 4. However, Clinpro's phosphate apposition effect had its greatest effect at 24 h in demineralized teeth and at 72 h in teeth with enamel defects (Figure 2). In contrast, studied plant extracts, in particular *P*. *marginatum* phosphate and orthophosphate apposition was not as important as that observed by Clinpro at 24 h (Figure 3). Furthermore, it was observed that *P*. *marginatum* extract signal lasted up to 48–72 h, suggesting extracts might contain secondary metabolites, similar to those identified in *Galla chinensis*, such as tannins, polyphenols, and flavonoids, which are capable of phosphate and orthophosphate apposition. Moreover, they prevent their dissolution in storage medium and maintain their concentration over time (Epasinghe et al., 2015; Kharouf et al., 2020; Petti & Scully, 2009).

Initial enamel composition observed changes at 0 h corresponded to enamel's mineral loss, due to phosphoric acid application as a demineralizing agent, resulting in what is known as an artificial dental caries or in vitro cavities. This phenomenon is observed with a decreased Raman intensity band corresponding to phosphate (955 cm^−1^) and orthophosphate (1037.5 cm^−1^), Figure 1a. Additionally, it was observed this mineral loss was not recovered to the initial levels, when teeth were stored for 6 months at 4°C in distilled water or artificial saliva (Figure 1b), neither was it recovered for the studied substances (*p* < 0.05). A similar behavior has been reported by Jain and collaborators (2019) in dental caries generated by tooth enamel demineralization by bacterial acid secretion (Streptococcus and Lactobacillus genera). In this process saliva is not capable of replacing these minerals causing a white spot phase, representing the clinically observable first phase of dental caries (Jain et al., 2019). In contrast, our AI results revealed statistically significant differences for the phosphate band in saliva stored teeth (E2) in comparison with teeth stored in distilled water (E1) (*p* = 0.047), suggesting saliva could contribute to phosphate apposition, due to the presence of phosphate ions in saliva.

*Ilex guayusa* and *P*. *marginatum* extract appositioned phosphate after the tooth was demineralized with a gradual increase, whereas for Clinpro the greatest apposition was at 24 h. The company that supplies this product, in its data sheet, indicates that remineralization is obtained 24 h after product application, this effect is lost as time progresses (ClinPro, 2015). Regarding orthophosphate apposition, significant differences were observed at 24 h between Clinpro and *I*. *guayusa*, with a higher activity for Clinpro. *I*. *guayusa* and *P*. *marginatum* extracts presented greater orthophosphate mineral apposition between 24 and 48 h. These results suggest the durability of these two extracts in the oral cavity for a longer time, preventing mineral loss, thus a greater apposition (Favaro et al., 2020). Our hypothesis proposes a possible protection effect against mineral dissolution, due to the presence of tannins and flavonoids, among others. This would represent a greater advantage in comparison with Clinpro. Moreover, its potential use would be centered especially in patients with great cavity susceptibility, and in patients with AI, who suffer from sensitivity to thermal changes.

Additionally, Recaldent™, is another remineralizing product commercially available and used in the common practice by health care professionals (America, 2020). Significant differences were observed for phosphate and orthophosphate during storage, suggesting some components in saliva could favor mineral apposition provided by this product. However, this effect was lower for Recaldent™ MI Paste in comparison with Clinpro and extracts. Recaldent™, did not exhibit the expected remineralization result in teeth with artificial cavities and AI. Authors, such as Zero (2009) indicate Recaldent™ demonstrated remineralization potential effect in their in situ laboratory results. However, this study is inconclusive and more studies are required to support Recaldent's™ commercial use (Zero, 2009). Furthermore, in the systematic review performed by Indrapriyadharshini and collaborators (2018) on CPP‐ACP, 12 articles out of 41 described a potential remineralizing effect on the CPP‐ACP complex when used at high concentrations (10%) in commercial products, such as GC Tooth mousse, MI Paste, and MI Paste Plus (Indrapriyadharshini et al., 2018). Nevertheless, in MI Paste Safety Data Sheet, CCP‐ACP is not listed, however, other compounds are, such as glycerol, carboxymethylcellulose, 1,2‐propanediol, and titanium dioxide (America, 2016). CPP is a milk derivative, which stabilizes phosphate in ACP, and thus the CCP‐AP complex, which releases phosphate and calcium ions required for tooth enamel remineralization (America, 2020; Reynolds, 1998; Rose, 2000). Additionally, when fluoride is added to products containing the CPP‐ACP complex, the final mineralization effect improves considerably (Shen et al., 2011).

It is noteworthy to mention that fluoride has been considered an efficient means in cavity prevention in its many commercial presentations, from water fluoridation schemes to mouthwashes, toothpastes, and varnishes. Fluoride action employs mechanisms, such as inhibiting demineralization, increasing mineral apposition on the surface of hydroxyapatite crystals, and decreasing crystal dissolution to the attacks of acids in the oral cavity. This interaction with hydroxyapatite increases crystalline stability and results in decreased tooth enamel solubility (Shen et al., 2011). Nevertheless, its toxicity has promoted the search of other equal and effective alternatives for remineralization, with fewer secondary effects (Tartaglia et al., 2019). Therefore, in the present work native plant extracts demonstrated the capacity to apposition phosphate and orthophosphate, possibly interacting with the tooth's enamel. Additionally, nowadays natural medications are preferred over conventional ones for various reasons, including low cost, availability, efficacy, and safety (Al‐Worafi, 2020; Dutra et al., 2016; Guevara et al., 2010).

This work is the initial phase on extract use in pathologies related to tooth enamel defects (AI). Therefore, it is recommended to analyze its possible toxicity, cytotoxicity, and viability for subsequent application in commercial product use. Likewise, topographic studies on the surface of dental tissue must be performed, to determine modifications that can result from extract use on the tooth's surface.

## 5 CONCLUSIONS

Raman spectroscopy technique is presented as a good research alternative for molecular structure, components, and molecule identification in Dentistry. Herein, it was possible to identify the main two components of tooth's enamel, phosphate, and orthophosphate in applied substances. From our data, we observed these extracts allowed phosphate and orthophosphate apposition with a lasting effect over time. In addition, *P*. *marginatum* had a better behavior in comparison to *I*. *guayusa*. Therefore, we suggest in the future extracts derived from native Colombian plants, such as *I*. *guayusa* Loe*s* and *P*. *marginatum* Jacq., could be used as new alternatives for tissue remineralization.

## CONFLICT OF INTEREST

The authors declare they have no competing interests.

## AUTHOR CONTRIBUTIONS

*Conception and design*: Sandra J. Gutiérrez‐Prieto, Luis G. Sequeda‐Castañeda. *Acquisition of data*: Gabriela M. Penedo‐Jaramillo, Andrea V. Chacín‐Nieto, Daniel R. Contreras‐Cáceres, María P. Galvis‐Rincón, Sandra J. Gutiérrez‐Prieto, Luis G. Sequeda‐Castañeda. *Analysis and interpretation of data*: Gabriela M. Penedo‐Jaramillo, Andrea V. Chacín‐Nieto, Daniel R. Contreras‐Cáceres, Gloria C. Moreno‐Abello, Sandra J. Gutiérrez‐Prieto, Luis G. Sequeda‐Castañeda. *Drafting the manuscript*: Gabriela M. Penedo‐Jaramillo, Andrea V. Chacín‐Nieto, Daniel R. Contreras‐Cáceres, Sandra J. Gutiérrez‐Prieto, Luis G. Sequeda‐Castañeda. *Revising the manuscript for intellectual content*: Fredy O. Gamboa‐Jaimes, Sandra J. Gutiérrez‐Prieto, Pilar E. Luengas‐Caicedo, Luis G. Sequeda‐Castañeda. *Final approval of the completed manuscript*: Sandra J. Gutiérrez‐Prieto, Pilar E. Luengas‐Caicedo, Luis G. Sequeda‐Castañeda.

## Data Availability

All data and materials used in this research are available from the corresponding author on reasonable request.
